# Heavy metal exposure risk associated with ingestion of *Oreochromis niloticus* and *Coptodon kottae* harvested from a lacustrine ecosystem

**DOI:** 10.1007/s10661-023-10936-0

**Published:** 2023-02-27

**Authors:** Awo Miranda Egbe, Pascal Tabi Tabot, Beatrice Ambo Fonge, Veronica M. Ngole-Jeme

**Affiliations:** 1 Department of Botany and Plant Physiology, University of Buea, PO BOX 63, Fako, Division South West Region Buea, Cameroon; 2 Department of Agriculture, Higher Technical Teachers’ Training College Kumba, Kumba, Meme Division, South West Region Cameroon; 3 Department of Environmental Sciences, College of Agriculture and Environmental Sciences, University of South Africa, Florida Campus, Roodepoort, Johannesburg, Gauteng Province 1710 South Africa

**Keywords:** *Coptodon kottae*, *Oreochromis niloticus*, Provisional tolerable weekly intake, Hazard quotient, Water quality index, Bio-sediment accumulation factor

## Abstract

Lacustrine ecosystems have not been widely assessed for heavy metal contamination and associated health risks; yet, they could be accumulating these contaminants to the detriment of aquatic organisms and communities relying on them for various aspects. The water quality index (WQI) and concentrations of heavy metals including As, Cd, Co, Cu, Cr, Fe, Mn, Ni, Pb, and Zn in water, sediment, *Oreochromis niloticus*, and in the endemic and endangered *Coptodon kottae* in Lake Barombi Kotto in Cameroon were determined to evaluate fish heavy metal bioaccumulation, and heavy metal exposure risk posed to communities consuming these fish species. The WQI of the lake was found to be excellent with heavy metal concentrations that were lower than what was obtained in the sediments and fish samples. Mean heavy metal concentrations in sediment ranged from 0.86 ± 0.03 mg/kg for Cd to 560.1 ± 11.15 mg/kg for Fe. In both fish species, Fe, Mn, and Cu had the highest concentrations. Though the heavy metal concentrations in the lake water were low, heavy metal bioconcentration factors for both fish species were very high ranging from 1.6 for Fe to 1568 for Mn. The concentration patterns of heavy metals in the organs of both fish species followed the order bones > gut > muscle. Consumption of these two fish species contributes less than 1.0% of the permissible tolerable daily intake (PTDI) and provisional tolerable weekly intake (PTWI) of these metals with lead (Pb) having the potential to exceed permissible exposure levels when high amounts of these fish are consumed by adults.

## Introduction

Aquatic environments are habitats for many organisms which interact with each other and abiotic factors in a self-perpetuating manner to ensure a dynamic and sustainable ecosystem. These environments play a major role in the sustenance of life in communities where they exist by providing water for consumptive and non-consumptive uses that include recreation, food, and medicinal herbs among others (Carpenter et al., 2011). They also filter sediments and pollutants from water and serve as buffer zones for flooding thereby maintaining water quality. Aquatic ecosystems, especially freshwater ecosystems, are, however, faced with challenges that include pollution and a reduction in the abundance of biodiversity (Bassem, 2020; Malik et al., 2020; Xu et al., 2014). Several chemical pollutants have been identified in aquatic environments, but heavy metals continue to present a significant threat and are recognized as major aquatic pollutants (Aboud & Nandini, 2009; Fernandez-Maestre et al., 2018). The sources of heavy metals in aquatic environments could be autochthonous, originating from natural processes like weathering around the catchment area, or allochthonous coming from atmospheric deposition, runoffs from agricultural fields, sewage discharge, industrial effluent discharge, dumping of waste, and boating activities among others (Algül & Beyhan, 2020; Boehnert et al., 2020; Fonkou et al., 2005).

Aquatic ecosystems including lakes are home to a variety of fish species that serve as a vital link in many aquatic food webs (Ersoy et al., 2019). Fish is also an important part of the human diet because of its high nutritional quality (Ayanda et al., 2018). It is a very good and reliable source of protein that is widely preferred to meat and other animal protein sources due to its low cholesterol content (Osman et al., 2001). Aquatic pollution however presents a significant challenge to these organisms. They are in direct contact with water and are therefore exposed to the contaminants that may be contained in the water. Fish in contaminated water bodies absorb dissolved substances including heavy metals directly from the water as it passes through their gills or through the ingestion of food that might have been contaminated with these metals. In addition to these two pathways of exposure, benthic feeders are likely to be exposed to contaminants through accidental ingestion of bottom sediments that may have adsorbed these contaminants, as bottom sediments serve as a sink for contaminants like heavy metals in aquatic ecosystems. Fish tend to bioaccumulate significant amounts of heavy metals from these abiotic sources.

Some heavy metals including iron (Fe), zinc (Zn), copper (Cu), manganese (Mn), and molybdenum (Mo) are essential in various metabolic processes, growth, and development of fish (Fawole et al., 2007), whereas others like lead (Pb), cadmium (Cd), nickel (Ni), and arsenic (As) play no vital role in these processes and are toxic even at very low concentrations. These metals also display a preference for specific organs. Lead and Cd, for example, have been found to accumulate in the gills, gut, muscles, and bones of fish to levels which can be hazardous to both the fish itself and other levels of the trophic chain, eliciting toxicological effects on reproductive cycles, shell formation, and other developmental processes (Adebayo, 2017; Authman et al., 2015; Ayanda et al., 2018; Pinzón-Bedoya et al., 2020). The content of toxic heavy metals in fish can counteract their beneficial effects on the consumer. Health risks associated with human exposure to heavy metals include renal failure, liver damage, cardiovascular diseases, carcinogenic, neurotoxic, and gastrointestinal ailments, and even death (Fernandez-Maestre et al., 2018; Ngole-Jeme & Fantke, 2017). Manifestation of these negative effects, however, depends on the level of exposure and the age of the consumer. Studies by Ngole-Jeme and Fantke (2017) have highlighted the differences in cancerous and non-cancerous risk associated with heavy metal exposure in adults and children.

Many international monitoring programs have been established to assess the quality of fish for human consumption and to monitor the health of the ecosystem in which they are found in an endeavor to prevent heavy metal bioaccumulation and biomagnification. Studies designed to monitor heavy metal pollution levels in aquatic ecosystems have mostly focused on surface water bodies that receive wastewater and industrial effluents, acid mine drainage, and those with catchment areas that include industrial and mining areas because these are documented sources of heavy metals to aquatic environments. A lot of these studies have also been directed towards dams, rivers, and streams, but information on pollution in lacustrine ecosystems is scanty. The quality of water in streams, dams, rivers, and lakes in areas with a subsistence lifestyle and where there are very little industrial activities is usually assumed. Lakes around the world provide more than 90 million tons of fish annually (Welcomme, 2011). The ever-increasing human population around this freshwater ecosystem threatens its productivity because of the increase in anthropogenic activities which tend to release pollutants like heavy metals that find their way into the lakes. Anthropogenic activities also contribute to the sediment load received by lakes through the acceleration of erosion rates. These sediments act as a sink for heavy metals in the lakes but may sometimes be the source of these same metals to the overlying water (Algül & Beyhan, 2020). Lake sediments could therefore be a point source of heavy metals to the water in the lake and to aquatic species there present.

This study uses Lake Barombi Kotto in Cameroon as an example of a lacustrine ecosystem which plays a significant role in the sustenance of life in surrounding communities. This lake is found along the Cameroon Volcanic Line and harbors at least eight different species of tilapia, including *Coptodon kottae* and *Oreochromis niloticus*. *Coptodon kottae* is endemic to lake Barombi Kotto and neighboring Lake Mboandong. Formerly called *Tilapia kottae*, *C. kottae* is a bentho-pelagic species of the cichlid family which is currently under threat from excessive sedimentation caused by deforestation of the surrounding riparian vegetation, pollution from agrochemicals, municipal waste, and sewage from surrounding settlements (Froese & Pauly, 2015). These fish species are widely consumed by surrounding communities, and so the presence of contaminants such as heavy metals in the lake may present a significant threat to these communities. This study determined the concentrations of Fe, Cu, Ni, Mn, Zn, Co, Ni, Pb, Cd, and Cr in the lake water, sediments, and the different organs of the fish species *C. kottae* and *O. niloticus* collected from Lake Barombi Kotto. These results were then used to evaluate the health risk associated with the consumption of these fish species by surrounding communities. The study also looked at the accumulation of heavy metals in the fish relative to that of the sediments and water in the lake and gives insight into the water quality of aquatic ecosystems in an area where there are few industrial sources of pollution. The outcome of this study provides strategic imperatives for the management of lakes and the exploitation of the resources therein.

## Materials and methods

### Description of the study area

**Fig. 1:**
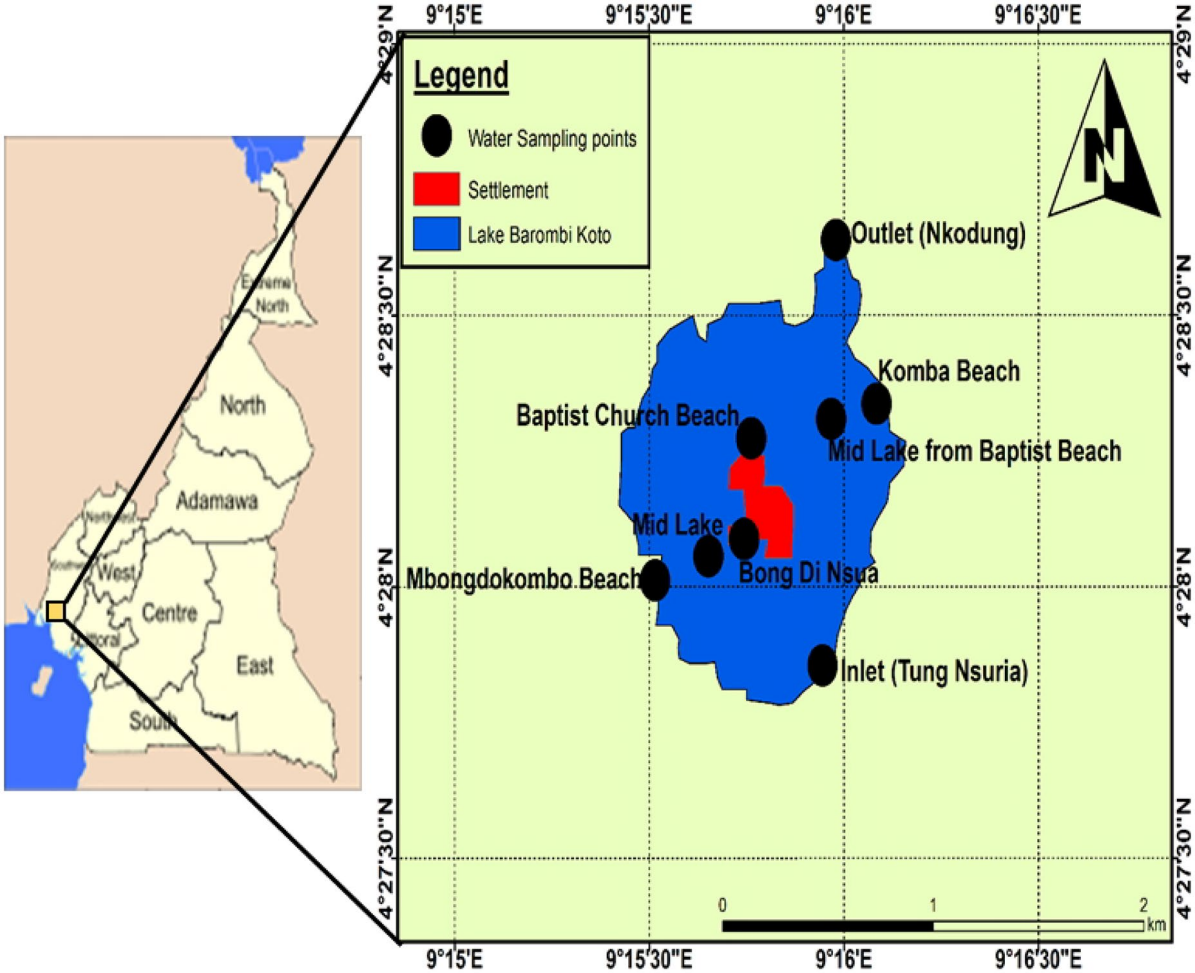
Location of sampling sites in Lake Barombi Kotto, Cameroon

Lake Barombi Kotto is a shallow freshwater crater lake located 100 m above sea level at latitude 4° 28′ N and longitude 9° 16′ E in Meme Division of the Southwest Region of Cameroon (Fig. 1). It has a total surface area of 330 hectares (3.3 km^2^), an average depth of 3.8 m, and a maximum depth of 6 m. The maximum length and width of the lake are 2.2 km and 2 km, respectively. The lake sits on a complex geological setting of basaltic volcanic rocks. Studies on the geochemistry of these rocks by Fitton and Dunlop (1985) revealed that they contain more than 4% of Mg and a relatively high content of trace elements which is released into the natural waters of the lake as these rocks weather. The lake harbors two islands; one of which is home to a large island community of the Barombi Kotto natives. The resident population impacts the lake through agricultural activities at the peripheries of the lake, laundry, construction, and improper sewage and refuse disposal. All run-off as well as silt from the surrounding agricultural farmlands and settlements empty into this lake (Awo et al., 2019). Fishing is the main occupation of the residents in the island. Previous studies on Lake Barombi Kotto by Awo et al. (2021), Awo (2020), and Campbell et al. (2017) have focused on health issues with respect to urogenital *Schistosomiasis*, water quality, and riparian tree diversity*.* No available literature that addresses the concentrations of heavy metals in the lake sediments and the fish harvested from the lake was found. The health risk associated with heavy metal exposure due to the consumption of fish from the lake is also not known.

### Sampling sites

Six different sites (S1, S2, S3, S4, S5, and S6) representing the different anthropogenic activities that could impact the quality of water in the lake were selected for sampling. These sites were Mbondokombo beach (S1), Bong di Nsua (S2), and Baptist Church (S3) which are located at the shore of the lake close to the settlement. The surrounding settlements have no sewage infrastructure, and so raw sewage is a potential source of pollutants at these sites. Komba beach where site S4 was located was close to agricultural farmlands in the area, whereas sites S5 (Tung) and S6 (Nkondong) were located at the inflow and outflow channels, respectively. Agricultural activities are the likely source of pollution at these sites. This lake is connected to River Meme through the outflow channel at site S6.

### Fish and sediment sample collection

Fish samples used in this study were bought from fishermen who harvest fish randomly from the lake using a fishing mesh commonly called cast net. Ten (10) samples each of *C. kottae* and *O. niloticus* were used in this study. The fish samples were washed with clean water, separated according to species, put in ice coolers, taken to the laboratory on the same day, and then frozen at − 20 °C until dissection. Fish samples were dissected using stainless steel scalpels. One gram (1 g) of accurately weighed epaxial muscle on the dorsal surface of each fish sample, the entire gut, and bones from each fish sample were collected for analysis. These fish organs were oven-dried in an open vented oven and powdered in the Life Sciences laboratory of the University of Buea in Cameroon.

Sediments from the lake were sampled at a depth of 0–5 cm using a Van Veen grab sampler at each of the six sampling sites, put immediately in airtight polyethene bags, and transported to the Life Sciences Laboratory at the University of Buea where they were air-dried and sieved for further analyses. All samples were collected in triplicates from each of the six sampling sites, making a total of 18 sediment samples. Both the dried fish and sediment samples were transported to the College of Agriculture and Environmental Sciences Laboratory of the University of South Africa in Johannesburg, South Africa for heavy metal analyses. Details of the water properties for both the wet and dry seasons of the lake have been published in Awo (2020), and so they were not determined in this study, but the values published by Awo (2020) were used in the determination of the water quality index (WQI) of the lake water.

### Heavy metals analyses of fish and sediment samples

In the determination of heavy metal content in the fish samples, 0.5 g of each of the dried and powdered fish sample organs were weighed into separate 50-ml microwave bomb digestion tubes, and 20 ml of concentrated HNO_3_ was added. The mixture was thoroughly swirled and allowed to stand for a few minutes at room temperature after which they were then microwave digested. Sediment samples were also microwave digested by adding aqua regia (HNO_3_ + 3HCl) in 1 g of each sample weighed into microwave digestion tubes. A Perkin Elmer Multiwave 3000 Microwave unit was used for digestion. The temperature of the microwave was raised to 160 °C over 10 min and held for 15 min at a power of 800 W and pressure of 30 bar. The digestion was carried out for 35 min at a rate of 0.5 bar s^−1^ (Lin et al., 2007). The digested samples were then cooled, filtered through acid-washed filter paper, and transferred into 50-ml volumetric flasks. The samples were made up to volume using distilled deionized water, and the concentrations of As, Cd, Co, Cu, Cr, Fe, Mn, Ni, Pb, and Zn in each digest were determined using a Perkin Elmer Nexion 300 Q ICP-MS.

### QC/QA measures

Sampling equipment and all glassware used for analyses were thoroughly washed with acidified water prior to their use. Analar grade reagents were used for all analyses. A Perkin Elmer Plus ICP multi-element standard was used in the calibration of the Perkin Elmer Nexion 300 Q ICP-MS used for analyses. To determine the percentage recovery and reliability of results of heavy metal concentration determined using the Perkin Elmer Nexion 300 Q ICP-MS, a solution with a known concentration of heavy metals prepared using the multielement standard was analyzed and the concentrations obtained from the equipment were compared with the known concentrations. All analyses were carried out in duplicate with two reagent blanks included in each batch of microwave digestion.

### Data analyses

#### Statistical analyses

Data on the heavy metal concentrations of the fish samples were analyzed statistically using the R-statistical software. Kruskal–Wallis rank sum test was used to compare and test for statistically significant differences between the respective organs of the two fish species: *O. niloticus* and *C. kottae*. Non-parametric tests were conducted since data were not normally distributed. Kruskal–Wallis rank sum test was preferred because after testing for normality of data, the distribution of most all the HM were skewed. However, when these data were transformed, using different transformation methods all passed the Kruskal–Wallis test for normality.

#### Determination of water quality of the lake

The means of water quality parameters of Barombi Kotto lake presented in Awo (2020) were used to determine the WQI of the river using Weight Arithmetic WQI as indicated in Eq. 1 (George & Ngole-Jeme, 2022).

1 $$WQI=\frac{\sum Wi \ qi}{\sum Wi}$$

where *WQI* is the water quality index, *i* is the number of water quality parameters, *qi* is the quality rating for each water quality parameter, and *Wi* is the relative weight for the water quality parameter. The quality rating (*qi*) for DO and pH was determined according to Eq. 2, whereas the *qi* for all other parameters was determined according to Eq. 3. The relative weights of the parameters were calculated using assigned water quality parameter weights (AW) presented by Kangabam et al. (2017) according to Eq. 4.

2 $$Qi\;(pH\;and\;DO=\left[\frac{V_{actual}-V_{ideal}}{Si-V_{ideal}\;}\right]\times100$$

3 $$qi=\frac{{V}_{\mathrm{actual}}}{Si}$$

4 $$Wi= \frac{AW}{{\sum }_{i=1}^{n}AW}$$

where *Qi* is the quality rating of the water quality parameter, *V*_actual_ is the actual value of the water quality parameter obtained from laboratory analysis, *V*_ideal_ is the ideal value of that water quality parameter that is assumed to be 7 for pH and 14.6 mg/l for DO, and *Si* is the WHO permissible level for the parameter.

The assigned weights for the different water quality parameters used were pH; 2.54, conductivity; 3.22, turbidity; 2.43, DO; 4.09, TDS; 2.75, total hardness; 1.46, NO_3_^−^; 2.57, NO_2_^−^; 2.00, P; 4.00, Na;1.67, Ca; 2.00, and Mg; 2.00 (Kangabam et al., 2017). These values were then used to determine the water quality index of the water in the Barombi Kotto lake where water with WQI values of 0–25 is considered excellent, 26–50 good, 51–75 poor, 76–100 very poor, and 100 and above unsuitable for drinking (Awachat & Salkar, 2017).

#### Determination of heavy metal accumulation by fish

The bioconcentration factor (BCF) and bio-sediment accumulation factor (BSAF) were used to determine the accumulation of heavy metals by both fish species as directed by Kwok et al. (2013). Whereas BCF was used to show how high the concentration of the heavy metals in the fish was relative to the lake water, BSAF was used to determine the intake of heavy metals by the fish from the sediments. The equations (Eqs. 5 and 6) proposed by Krivokapić (2021) and Kwok et al. (2013) were used in the determination of BCF and BSAF, respectively.

5 $$BCF= \frac{\mathrm{concentration \ of \ heavy \ metal \ in \ fish \ organ}}{\mathrm{concentration \ of \ heavy \ metal \ in \ the \ water}}$$

6 $$BSAF= \frac{\mathrm{Metal \ concentration \ in \ fish \ organ}}{\mathrm{metal \ concentration \ in \ sediment}}$$

#### Determination of heavy metal exposure risk associated with consumption of fish

Screening-level human toxicity assessment of all heavy metals was done using characterization factors of the USEtox Model (Rosenbaum et al., 2008). The USEtox Model is a model developed through the UNEP-SETAC Life Cycle initiative that is used to characterize human and ecological impacts in life cycle assessments (Hauschild et al., 2008; Rosenbaum et al., 2008). The USEtox Model combines the fate of chemicals of interest in various environments, exposure to human through various routes, as well as the potential of the metals to cause a negative effect in a set of characterization factors that are then used to determine the health risk they pose to humans and ecosystems. In this study, USEtox Model characterization factors for the emission of As, Cr, Cd, Co, Cu, Fe, Mn, Ni, Pb, and Zn into freshwater ecosystems in North, West, East, and Central Africa were contrasted, and the contribution of ingestion and inhalation exposure pathways to the overall human exposure was considered as a first step towards the determination of the health risk associated with the metals considered in this study (Ngole-Jeme & Fantke, 2017). These analyses complimented the Risk Assessment guidance for superfund Volume I Human Health Evaluation Manual Part A (Qu et al., 2012; USEPA, 2010).

In determining the health risk associated with heavy metal exposure through the consumption of both fish species from Lake Barombi Kotto, USEPA guidelines as indicated in Qu et al. (2012), and USEPA (2010) were used. The daily intake of the heavy metals by individuals ingesting fish from the lake was determined using Eq. 7 (Gbogbo et al., 2018; Kar et al., 2011; Pinzón-Bedoya et al., 2020).

7 $$ADI= \frac{MC \ {x} \ DI \ {x} \ CF \ {x} \ EF \ xED}{BW \ {x} \ AT}$$

where ADI, MC, DI, EF, ED, AT, and BW represent average daily intake (mg/kg body weight /day), metal concentration in fish (mg/kg), daily intake of fish (mg/day), exposure frequency (days/year), exposure duration (years), average time of exposure (days), and individual body weight (kg), respectively. The values used for these various parameters are included in Table 1.

**Table 1:** Parameters used in the calculation of health risks

Parameters	Unit	Definition	Values used	
			Adult	Children
ADI	mg/kg body weight/day	Average daily intake of heavy metal		
MC	mg/kg	Metal concentration	As presented in Table 3	
DI	mg/day	Daily intake of fish	52	52
BW	kg	Body weight	70	25
R*f*D	mg/kg/day	Oral reference dose of metal	As = 3.0 × 10^−4^, Cd = 1 × 10^−3^ Co = 3.0 × 10^−3^, Cr = 1.5 × 10^−3^, Cu = 4.0 × 10^−2^, Fe = 7 × 10^−1^, Mn = 4.6 × 10^−2^ Ni = 2.0 × 10^−2^, Pb = 3.5 × 10^−5^ Zn = 3.0 × 10^−1^	
*EF	Days/year	Exposure frequency	52 208 364	52 208 364
ED	Years	Exposure duration	65	6
AT	Days	Average time over which exposure is averaged	ED X 365	ED X 365
CSF	mg/kg/day	Carcinogenic slop factor	As = 1.5, Cd = 3.8 × 10^−1^ Ni = 0.84, Pb = 8.5 × 10^−3^	

^*^Exposure frequency considered were daily (365 days/year), 4 times a week (208 days/year), and once a week (52 day/year)

The metal concentration used for the determination of health risk was the mean of the metal concentration obtained from 10 different fish samples of each species analyzed. Results of studies on food consumption in Cameroon by Pouokam et al. (2017) put the average consumption of freshwater fish at 52 g/day, but Pouomogne and Pemsl (2008) also indicated values as low as 15 g/day in villages and up to 95 g/day in larger cities of the country. These values were therefore used in the determination of average minimum and maximum daily intake of heavy metals through ingestion of *C. kottae* and *O. niloticus* from the lake. The average body weight was taken to be 70 kg for an adult and 25 kg for children. Values of ADI were then used to determine the hazard quotient (HQ) of the different metals as indicated in Eq. 8.

8 $$HQ= \frac{ADI}{RfD}$$

where *RfD* represents the reference dose for oral intake of the respective metals (mg/kg BW/day). The reference dose is the maximum acceptable amount of a metal that can be taken in a day without any health threats.

According to Pinzón-Bedoya et al. (2020), HQ is used to represent the risk of a heavy metal relative to its reference dose. HQ values greater than 1 indicate a potential exposure risk, whereas values below 1 indicate no exposure risk (Ngole-Jeme & Fantke, 2017; Qu et al., 2012). Reference doses used for the heavy metals were according to values presented in the United States Environmental Protection Agency (USEPA, 2017) and the Agency for Toxic Substances and Disease Registry (ATSDR, 2021). These values are also included in Table 1. Carcinogenic health risks associated with the consumption of the two species of fish from the lake were determined as indicated in Eq. 9.

9 $$CR= ADI \ {x} \ CSF$$

where *CSF* is the carcinogenic slope factor for each metal through the oral exposure route. Details of the values used in Eqs. 7–9 are presented in Table 1.

The estimated daily and weekly intake of the different heavy metals was also determined. Values for the estimated weekly intake (EWI) of heavy metals through fish consumption were determined for three different scenarios: consumption of fish once a week, four (4) times a week, and daily (7 times a week). To obtain the EWI of heavy metals by individual ingesting fish from the study area, the ADI values were multiplied by seven. These values were then compared with the recommended provisional tolerable weekly intake (PTWI) values for the respective metals to determine the percentage of the PTWI for each metal that is contributed by fish consumption. The PTWI estimates the maximum amount per unit body weight of a potentially harmful substance or contaminant in food or water that can be ingested weekly without risk of adverse health effects. The PTWI values used for the different toxic metals have been published in the JECFA guidelines of 2003 and the FAO/WHO expert committee on food additives (FAO/WHO, 2010). According to this guideline, the permissible tolerable daily intake (PTDI) values for the different metals are 7, 5.6, 3.5, 2–5, 0.05–0.2, 0.035, 0.025, and 0.007 mg/kg for Zn, Fe, Cu, Mn, Cr, Ni, Pb, and Cd, respectively, which is equivalent to PTWI of 490, 392, 245, 140–350, 3.5–14, 2.45, 1.75, and 0.49 mg/week, respectively, for a 70 kg adult person.

## Results and discussion

### Water quality of the Barombi Kotto lake

Except for phosphorus (P), the properties of water in the lake were all within the WHO stipulated limits for drinking water (Table 2). The source of P in the stream could be agricultural activities, the disposal of waste, and the leaching of sewage from nearby settlements into the lake. Studies by Bowes et al. (2015), Bunce et al. (2018), and Gall et al. (2015) have all highlighted agricultural activities as a major source of P to many water bodies. The high concentration of P may indicate a potential for the development of eutrophic conditions in the lake, with consequences for organisms inhabiting the lake and communities which rely on it for the provision of water for various purposes. Analyses of the water quality of the lake using pH, conductivity, turbidity, DO, TDS, total hardness, NO_3_^−^, NO_2_^−^, P, Na, Ca, and Mg yielded a WQI value of 11.80 which according to Kangabam et al. (2017) and Awachat and Salkar (2017) can be classified as excellent for drinking purposes despite the high P content of the water. Intake of high P content may rarely be manifested clinically, but some studies have reported significant health threats associated with excess P intake in individuals. In recent times, intake of excess P has been incriminated in phosphate, calcium, and vitamin D regulation with consequences on mineral metabolism and chronic kidney disease (Moe et al., 2006), vascular disease (Dhingra et al., 2007; Tonelli et al., 2005), and bone loss (Calvo & Uribarri, 2013). Considering that P is contained in many other foodstuffs consumed, monitoring of blood P levels in individuals making use of water from this lake for domestic purposes may be necessary to avoid negative health outcomes. Though the determination of WQI of the Barombi Kotto lake waters did not take into consideration the concentrations of heavy metals, their values in the lake were all below the WHO maximum acceptable limits for drinking water (Table 2). Previous studies by Awo (2020) indicated that the heavy metal pollution load index of the river was 6.2 in the rainy season and 4.7 in the dry season which are all lower that the critical pollution level according to Prasad and Maiti (2016). The heavy metal pollution levels of the river water are therefore very low.

**Table 2:** Water quality parameters of Barombi Kotto lake

Parameter	Mean	Standard error	Median	Standard deviation	Range	Minimum	Maximum	WHO standards	
pH	8.18	0.30	8.2	0.61	1.10	7.60	8.70	6.5–8.5	
Conductivity (µS/cm)	151.55	22.37	141.6	44.75	97.60	112.70	210.30	1500	
Temperature (°C)	23.05	0.38	23.2	0.75	1.80	22.00	23.80	25	
Total hardness CaCO_3_	39.03	1.02	38.55	2.04	4.80	37.10	41.90	-	
DO (mg/l)	2.30	0.06	2.3	0.12	0.20	2.20	2.40	5	
TDS (mg/l)	500.75	0.30	0.35	0.59	1.30	0.00	1.30	500	
P (mg/l)	7.48	1.25	7.5	2.50	5.90	4.50	10.40	0.1	
NH_4_^+^ mg/l)	2.10	0.38	1.85	0.76	1.70	1.50	3.20	-	
NO_3_^−^ (mg/l)	2.18	0.32	2.15	0.64	1.40	1.50	2.90	50	
NO_2_^−^ (mg/l)	1.70	0.18	1.7	0.37	0.80	1.30	2.10	3	
HCO_3_^−^ (mg/l)	25.98	1.30	25.65	2.61	5.80	23.40	29.20	-	
Ca (mg/l)	11.90	0.80	12.6	1.61	3.40	9.50	12.90	700	
Mg (mg/l)	2.58	0.51	2.6	1.01	1.90	1.60	3.50	150	
K (mg/l)	2.93	0.65	2.85	1.30	2.40	1.80	4.20	12	
Na (mg/l)	0.55	0.12	0.55	0.24	0.50	0.30	0.80	50	
Fe (mg/l)	3.73	0.59	3.55	1.17	2.40	2.70	5.10	0.3	
Zn (mg/l)	0.02	0.00	0.02	0.00	0.00	0.02	0.02	15	
Cu (mg/l)	0.01	0.00	0.01	0.00	0.01	0.01	0.02	2	
Mn (mg/l)	0.00	0.00	0.002	0.00	0.00	0.00	0.00	0.01	
Pb (mg/l)	0.00	0.00	0.001	0.00	0.00	0.00	0.00	0.01	
Cd (mg/l)	0.00	0.00	0.001	0.00	0.00	0.00	0.00	0.003	

Modified after (Awo, 2020)

### Heavy metal concentration in sediments

**Fig. 2:**
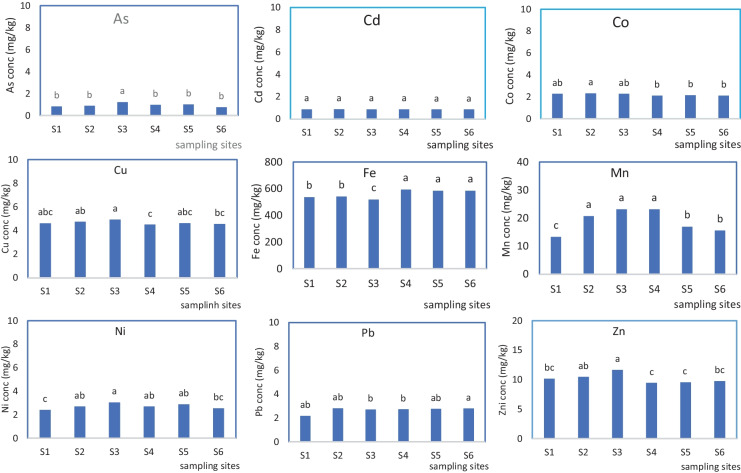
Concentrations of heavy metals in the sediments at Lake Barombi Kotto

Metal recovery using the multielement standard ranged from 82.8 to 107.9% with the highest percentage recovery recorded for Zn and the lowest for As. The concentration of the heavy metals in the sediments was in the order: Fe > Mn > Zn > Cr > Cu > Ni > Pb > Co > As > Cd (Fig. 2). The high Fe content of these sediments relative to the other elements studied could be attributed to the basaltic nature of soils in this area. Basalts are enriched in Fe and Fe family elements like Ti, V, Co, and Ni. In addition, Manga et al. (2013) have indicated that increasing weathering intensity in this area results in a decrease in silica content in soils in the area while oxides of Fe and Al accumulate, which could explain the high content of Fe in the sediments in the lake. The metal concentrations in sediments varied from one site to the other as indicated by the mean separators in Fig. 2.

Heavy metal concentrations in the lake sediments were significantly higher than what was obtained in the lake water. This is in line with reports by Chapman et al. (2011), Weber et al. (2013), and Cicero-Fernández et al. (2017) who all indicated that heavy metals in aquatic environments tend to be sorbed onto the sediments at the bottom of the water body from where they could be immobilized if chemical conditions in the water body change. In addition, the high concentrations of phosphorus in the water coupled with the slightly alkaline conditions could result in the precipitation of these metals in the bottom sediments as insoluble phosphates resulting in the higher concentration of the metals in the sediments compared to the water.

### Concentrations of heavy metals in fish species

**Fig. 3:**
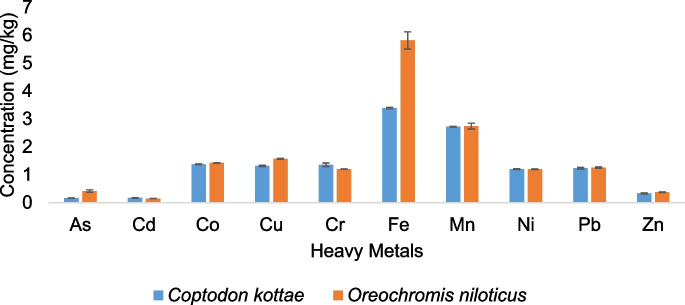
Total heavy metal concentration in fish

All 10 heavy metals investigated in this study were detected in both fish species as shown in Fig. 3. In both species, Fe, Mn, and Cu had the highest concentration, whereas As and Ni had the lowest (Fig. 3). A similar pattern of metal concentration was recorded in both fish species. In *C. kottae*, the concentration pattern was Fe > Mn > Co > Cr > Cu > Pb > Ni > Zn > Cd > As, and in *O. niloticus*, it was Fe > Mn > Cu > Co > Pb > Cr > Ni > As > Zn > Cd (Fig. 3). Similar findings were reported by Adebayo (2017) who found high levels of Fe and Zn in *Hemichromis faciatus* in Ureje, Lagos. Except for Pb in both species where the concentration in both fish species was higher than the World Health Organization (WHO) guidelines (0.5 mg/kg dry weight) and FAO guidelines (2 mg/kg dry weight), the concentrations of heavy metals in the fish were mostly within acceptable limits.

The high content of Fe, Mn, and Cu in the tissues of both fish species could be attributed to their metabolic necessities and the high intake of plankton present in the bottom sediments which constitute their food. Metal accumulation in fish depends on numerous factors such as food habits of the fish, trophic status, source of the metal in question, distance of the fish from the contamination source, and the presence of other ions in the environment. The concentrations of the heavy metals in the fish were much higher than what was obtained in the water from the lake but lower than the heavy metal concentrations in the sediments. Similar results were obtained by Weber et al. (2013) in a subtropical Brazilian river and were also confirmed by analyses of the BCF and BSAF carried out in this study.

**Fig. 4:**
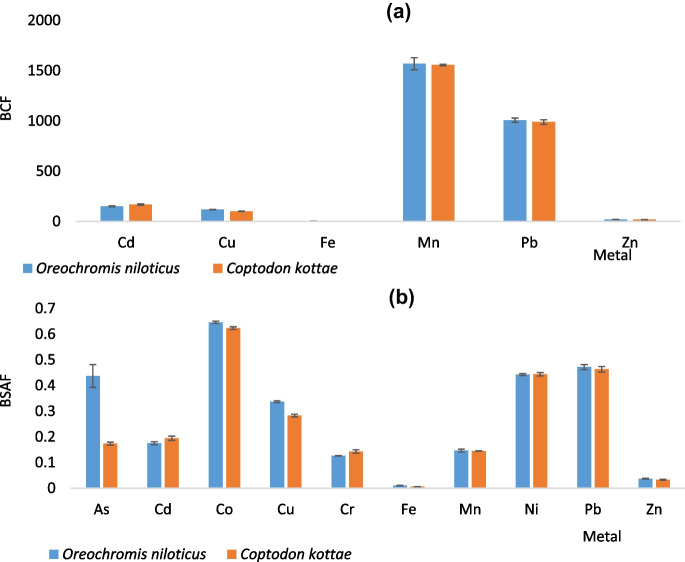
Bioconcentration factor (**a**) and bio sediment accumulation factor (**b**) of heavy metals by both fish species

Very high bioaccumulation of all heavy metals except Fe and Zn was observed in both fish species from the water (Fig. 4a). However, both species accumulated similar amounts from the water though BCF differed among metals (*P* = 0.001). The accumulation of heavy metals by the fish from the lake water was significantly higher than the accumulation from the sediments (*P* = 0.00) as indicated in Fig. 4b.

Similarly, differences observed in the accumulation of the various heavy metals from the sediments were significant (*P* = 0.00). Apart from As where *O. niloticus* accumulated more from the sediment (Fig. 4b), no difference was observed between the amount of metals accumulated by the two fish species. These findings are similar to what Hossain et al. (2022) found out in their study in terms of variation of bioaccumulation of different heavy metals by fish species but differ in the aspect of variation with fish species as bioaccumulation of each metal was similar for both species except for As (Fig. 4b). This study highlights the ability of fish to bioaccumulate heavy metals even if the concentrations of the metals in the abiotic environment where the fish is found are low. According to Ahmad and Al-Mahaqeri (2015), fish occupies a higher trophic level and tends to accumulate heavy metals from food, water, and sediments, which could account for the high bioaccumulation observed. The two fish species used in this study are also bentho-pelagic species which are likely to scavenge for food from bottom sediments and suspended particulates in the lake waters, both of which are known to adsorb heavy metals from interstitial waters. They are therefore exposed to heavy metals directly through absorption on the gills as they respire and ingestion of food items that may have accumulated heavy metals, and indirectly through accidental ingestion of sediments as they scavenge for food in the bottom sediments. The higher concentrations of the heavy metals in the fish relative to the water are therefore explained.

Whereas most studies by Bawuro et al. (2018) and Jezierska and Witeska (2006) have reported high bioaccumulation of Zn and Fe, these two metals were the least accumulated by both fish species. The pattern of heavy metal accumulation by both fish species from the lake water followed the order Mn > Pb > Cd > Cu > Zn > Fe. Whereas from sediments, the pattern was Co > Pb > Ni > Cu > As > Cd > Cr = Mn > Zn > Fe. Differences in patterns suggest that accumulation of heavy metals is not directly proportional to the heavy metal concentrations to which the fish are exposed but may vary with other factors among which are selective absorption of metals, rates of uptake, storage, and elimination (Tüzen, 2003). These results indicate that these fish species do not accumulate Fe and Zn from the abiotic environment but take up significant amounts of Mn and Pb from the water and Co, Pb, and Ni from the sediments.

### Distribution of heavy metals in fish organs

**Fig. 5:**
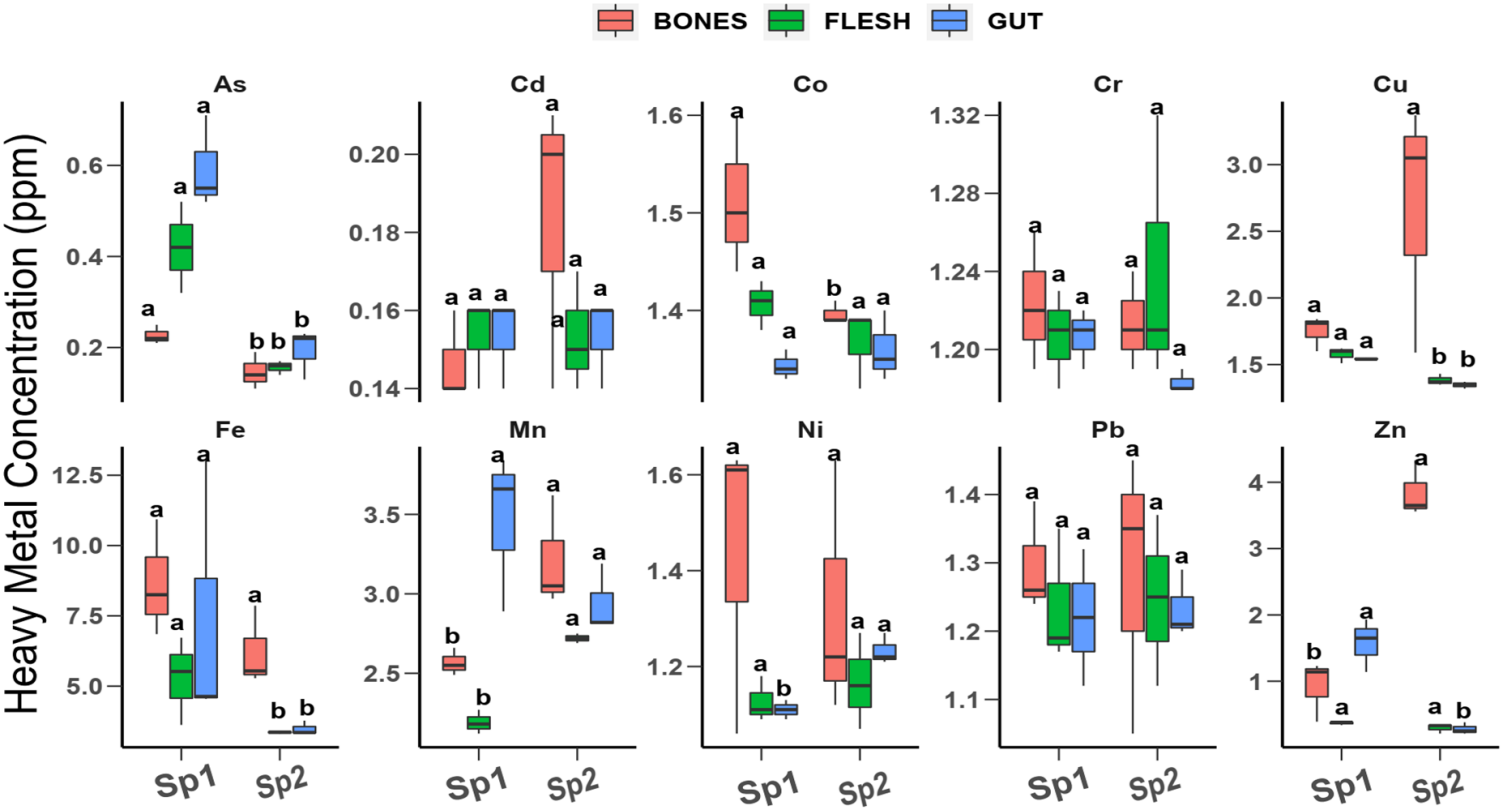
Comparisons of heavy metal concentration in organs (bones, flesh, and gut) of *Oreochromis niloticus* (Sp1) and *Coptodon kottae* (Sp2). Letters above the boxes indicate the level of significance according to the Kruskal–Wallis test. For a pair of organs, boxes with similar letters are not significantly different

The muscles, gut, and bones of *C. kottae* and *O. niloticus* accumulated different amounts of the heavy metals investigated (Fig. 5). The bones of both *O. niloticus* and *C. kottae* recorded significant amounts of Fe (8.67 ± 4.08 and 6.22 ± 2.5 mg/kg), Mn (2.56 ± 0.17 and 3.21 ± 0.67 mg/kg), Zn (0.92 ± 0.84 and 3.84 mg/kg), and Cu (1.75 ± 0.2 and 2.67 ± 0.78 mg/kg), respectively. The concentrations of non-essential metals including As, Cd, Co, Cr, Ni, and Pb in the bones of the fish ranged from 0.12 to 1.4 mg/kg with no significant differences in the concentrations of As, Co, Mn, and Zn in the bones of the two fish species (Fig. 5). Copper Fe and Zn accumulated more in the bones of *C. kottae*, whereas for *O. niloticus*, it was Mn that accumulated more in the bones. The percentage of the total of each metal concentration in the bones of both fish species ranged from 10.7% for As to 53.27% for Mn in *O. niloticus* and 33.42% for Pb to 87.3% for Zn in *C. kottae.* Very few studies have investigated heavy metal concentrations in the bones of fish, but results from this study indicate that fish bones were good accumulators of the heavy metals. Given that the bones of these fishes are sometimes chewed alongside muscles when being eaten, fish bone ingestion could be a hidden exposure pathway of heavy metals to consumers with a passion for chewing fish bones.

**Fig. 6:**
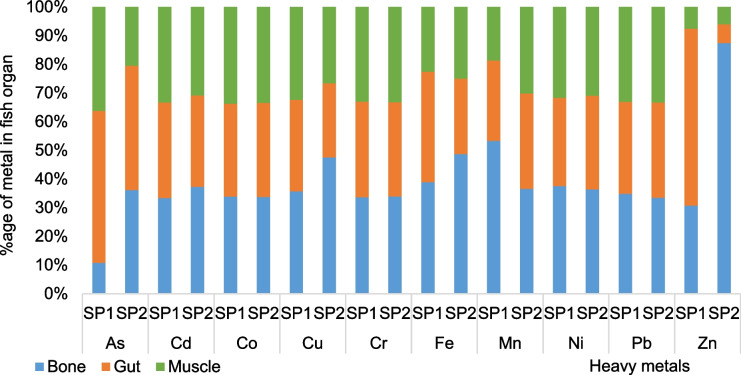
Partitioning of heavy metals into the bone, gut, and muscle of *Oreochromis niloticus* (Sp1) and *Coptodon kottae* (Sp2)

The concentrations of heavy metals in the gut of *O. niloticus* followed the order Fe > Mn > Cu = Zn > Co > Cr = Pb > Ni > As > Cd (Fig. 6). A different pattern of heavy metal concentration was obtained in the gut of *C. kottae* where the concentration pattern was Fe > Mn > Co = Cu > Pb = Ni > Cr > Zn > As > Cd as indicated in Fig. 6. There were significant differences in the concentrations of As, Cu, Fe, Ni, and Zn in the gut of *C. Kottae* and *O. niloticus* (Fig. 6). The percentage accumulation of metals in the gut of the fish ranged from 28.08% for Mn to 61.65% for Zn in *O. niloticus* and 6.5% for Zn to 43.4% for As in *C. kottae* (Fig. 6). Arsenic in both species accumulated more in the gut, whereas Zn preferred the gut of *O. niloticus* (Fig. 6).

Significant differences were observed in the concentrations of As, Cu, Fe, Mn, and Zn in the muscles of both fish species (Fig. 6). The metal with the highest concentration in the muscle of *O. niloticus* and *C. kottae* was Fe with concentrations of 5.2 mg/kg and 3.4 mg/kg, respectively. Arsenic had the lowest concentration in the muscle of *C. kottae* (0.09 mg/kg), whereas for *O. niloticus*, the metal with the lowest concentration in the muscle was Cd (0.15 mg/kg). Percentage accumulation in the muscle of the fish ranged from 7.63% for Zn to 36.31% for As in *O. niloticus* and from 6.09% for Zn to 33.45% for Co in *C. kottae* (Fig. 6).

The preferential segregation of the metals to the gut, bones, and muscle of the two fish species varied with fish species. Except for As and Zn in *O. niloticus* where the metal concentration pattern in organs was gut > muscle > bone and gut > bone > muscle, respectively, the concentration of the other metals in the different organs followed the pattern bone > muscle > gut (Fig. 6). Arsenic and Pb concentrations in the organs of *C. kottae* followed the order gut > bone > muscle, whereas the other metals had the same pattern as what was observed in *O. niloticus* (bone > muscle > gut). Several studies have reported low metallic concentration in muscles in relation to other organs. Studies by Qadir and Malik (2011), El-Moselhy et al. (2014), and Bawuro et al. (2018) indicated metal concentration patterns in fish to be in the order liver > gills > kidneys > muscles. This pattern, however, varies with the metal in question. Hossain et al. (2022) have shown that Cu, Zn, Fe, and Cd will preferentially accumulate in fish liver, whereas Pb and Mn seem to prefer fish gills. According to Qadir and Malik (2011) and El-Moselhy et al. (2014), the segregation of Pb, Cd, Cr, and Cu in various fish species follows the order liver > gills > kidneys > muscles. The gills and gut of fish are expected to be the main route of entry of heavy metals into the fish with the gills absorbing metals dissolved in the water and the gut absorbing the metals present in food and particulates ingested by the fish. Heavy metal concentrations in the gut may represent heavy metals that are absorbed through food and particulates ingested (Dallinger et al., 1987).

### Human exposure to heavy metals due to fish consumption

**Fig. 7:**
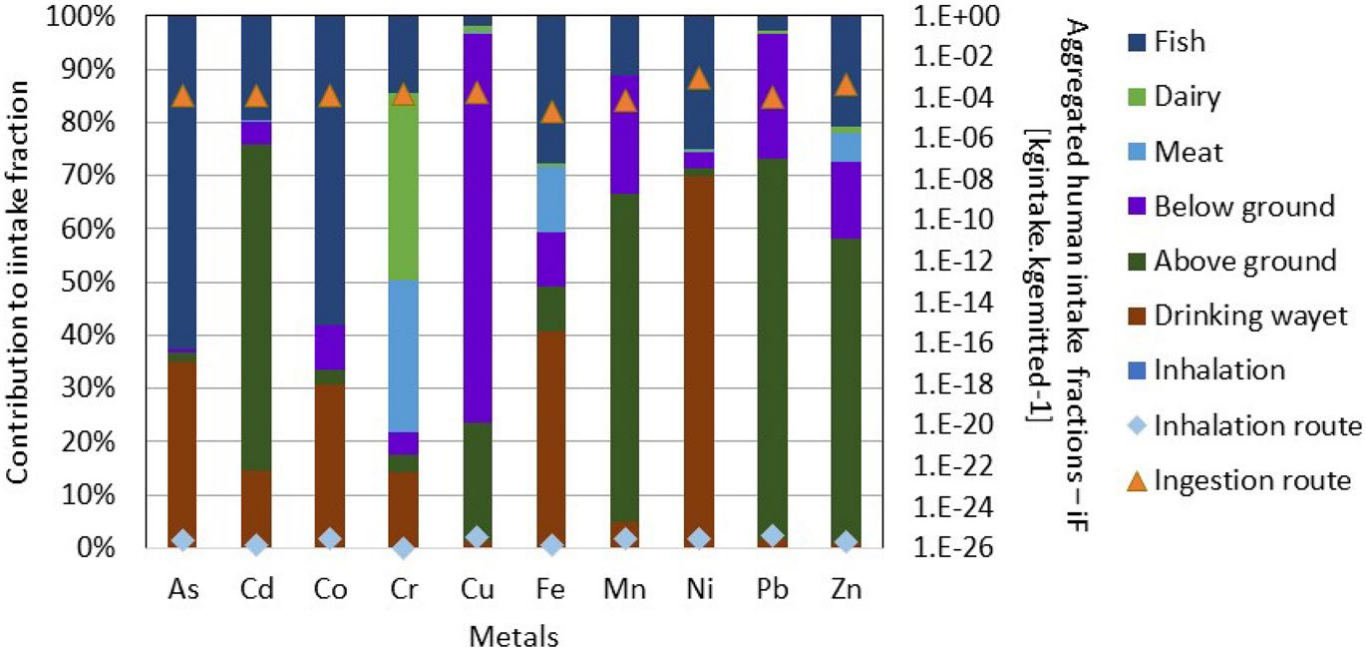
Heavy metal pathways that contribute to potential human exposure following an assumed exposure in freshwater ecosystems

Results from screening-level human toxicity for the heavy metals using the USEtox Model indicated that for metals emitted into freshwater ecosystems, exposure through the ingestion route which may include fish, below and above ground produced crops, drinking water, dairy, and meat (Fig. 7) are significantly higher than exposure that may occur via inhalation for all the heavy metals investigated. Dermal absorption is another route of exposure, but the USEtox Model does not take into consideration this pathway of exposure, and so it was not considered in this screening-level assessment. Whereas exposure to all the heavy metals may occur through the ingestion of fish and water, Cd, Mn, Pb, and Zn exposure are likely to occur mainly through the ingestion of crops grown above ground that have possibly been irrigated with water from these ecosystems (Fig. 7). Fish ingestion contributes up to 55% of human exposure to As and Co with drinking water contributing between 30 and 70% of exposure to As, Co, Fe, and Ni (Fig. 7). The least exposure pathway was observed for meat and dairy ingestion and inhalation. Chromium according to the screening-level assessment has a slightly different pattern where dairy and meat ingestion was the main exposure pathway. This according to Ngole-Jeme and Fantke (2017) is because of the affinity of Cr especially trivalent Cr to organic matter.

Analyses of human health risk associated with exposure through fish ingestion indicate that, except for Ni and Pb, values for adult and child ADI of heavy metals from consumption of *O. niloticus* were higher than ADI values associated with the consumption of *C. kottae.* This could be an indication that consumption of *O niloticus* presents more heavy metal exposure risk to individuals than *C. Kottae* (Table 3). This risk also increases with the average amount of fish ingested in a day as is expected. Considering that individuals from larger cities are the ones with an average intake of 95 g of fish daily, the heavy metal exposure risk is higher in cities than in villages. Among the heavy metals, values for ADI followed the order Fe > Mn > Co > Cu > Cr = Ni = Pb > Zn > As > Cd (Table 3). The daily and weekly tolerable intake of heavy metals by children was not considered in this study as no PTDI and PTWI values for children are available. Only those for the adults were considered. Consumption of either fish species by adults accounts for less than 1.0% of the PTDI of all the heavy metals studied except for Co (Table 4). Similarly, the estimated weekly intake values for the different heavy metals from the consumption of both fish species contribute less than 0.5% of the PTWI. The highest contribution made by fish to the PTWI was for Co, whereas the lowest contribution was for Zn. Consumption of *C. kottae* contributed more to the PTWI of most of the metals than *O. niloticus* (Table 4). As low as this contribution is, it may become significant should the other food items ingested by the adults have high concentrations of these heavy metals.

**Table 3:** Average daily intake of heavy metals by adults and children and the respective hazard quotients

Fish species	Heavy metal	Daily intake (mg/kg bw/day)						Hazard quotient (HQ)					
		15 gm/day		52 g/day		95 g/day		15 gm/day		52 g/day		95 g/day	
		Adult	Child	Adult	Child	Adult	Child	Adult	Child	Adult	Child	Adult	Child
*Oriochromianiloticus*	As	1.27E-05	1.09E-09	4.4E-05	1.3E-08	8.04E-05	4.35E-08	0.042333	3.62E-06	0.146753	4.35E-05	0.268107	0.000145
	Cd	4.58E-06	3.91E-10	1.59E-05	4.7E-09	2.9E-05	1.57E-08	0.004579	3.91E-07	0.015875	4.7E-06	0.029002	1.57E-05
	Co	4.35E-05	3.72E-09	0.000151	4.47E-08	0.000276	1.49E-07	0.014501	1.24E-06	0.05027	1.49E-05	0.09184	4.97E-05
	Cr	3.68E-05	3.15E-09	0.000128	3.78E-08	0.000233	1.26E-07	0.024545	2.1E-06	0.085089	2.52E-05	0.15545	8.42E-05
	Cu	4.79E-05	4.09E-09	0.000166	4.92E-08	0.000303	1.64E-07	0.001197	1.02E-07	0.004149	1.23E-06	0.007579	4.10E-06
	Fe	0.000178	1.52E-08	0.000615	1.82E-07	0.001124	6.09E-07	0.000254	2.17E-08	0.000879	2.61E-07	0.001606	8.70E-07
	Mn	8.38E-05	7.16E-09	0.000291	8.61E-08	0.000531	2.87E-07	0.001822	1.56E-07	0.006315	1.87E-06	0.011538	6.25E-06
	Ni	3.66E-05	3.13E-09	0.000127	3.76E-08	0.000232	1.26E-07	0.001832	1.57E-07	0.00635	1.88E-06	0.011601	6.28E-06
	Pb	3.84E-05	3.28E-09	0.000133	3.95E-08	0.000243	1.32E-07	1.097277	9.38E-05	3.803894	0.001127	6.949421	0.003762
	Zn	1.14E-05	9.79E-10	3.97E-05	1.18E-08	7.25E-05	3.93E-08	3.82E-05	3.26E-09	0.000132	3.92E-08	0.000242	1.31E-07
*Coptodonkottae*	As	5.04E-06	4.31E-10	1.75E-05	5.17E-09	3.19E-05	1.73E-08	0.016791	1.44E-06	0.058207	1.72E-05	0.106341	5.76E-05
	Cd	5.10E-06	4.36E-10	1.77E-05	5.24E-09	3.23E-05	1.75E-08	0.005098	4.36E-07	0.017674	5.24E-06	0.032289	1.75E-05
	Co	4.20E-05	3.59E-09	1.5E-04	4.31E-08	0.000266	1.44E-07	0.013992	1.20E-06	0.048506	1.44E-05	0.088617	4.80E-05
	Cr	4.15E-05	3.55E-09	1.444–04	4.27E-08	0.000263	1.42E-07	0.027679	2.37E-06	0.095954	2.84E-05	0.175301	9.49E-05
	Cu	4.02E-05	3.44E-09	1.39E-04	4.13E-08	0.000255	1.38E-07	0.001006	8.6E-08	0.003487	1.03E-06	0.006371	3.45E-06
	Fe	0.000103	8.85E-09	0.000359	1.06E-07	0.000655	3.55E-07	0.000148	1.26E-08	0.000513	1.52E-07	0.000936	5.07E-07
	Mn	8.31E-05	7.11E-09	0.000288	8.54E-08	0.000526	2.85E-07	0.001807	1.54E-07	0.006265	1.86E-06	0.011445	6.20E-06
	Ni	3.67E-05	3.14E-09	0.000127	3.77E-08	0.000233	1.26E-07	0.001836	1.57E-07	0.006366	1.89E-06	0.01163	6.30E-06
	Pb	3.77E-05	3.22E-09	0.000131	3.87E-08	0.000239	1.29E-07	1.077216	9.21E-05	3.734347	0.001107	6.822365	0.003693
	Zn	1.01E-05	8.64E-10	3.5E-05	1.04E-08	6.4E-05	3.46E-08	3.37E-05	2.88E-09	0.000117	3.46E-08	0.000213	1.15E-07

**Table 4:** Estimated daily and weekly intakes of heavy metal associated with the consumption of the muscles of *Oreochromis niloticus* and *Coptodon kottae*

Fish species	Heavy metals	PTDI	PTWI	ETDI		ETWI		Percentage contribution of ETDI to PTDI	Percentage contribution of ETWI to PTWI
				Minimum	Maximum	Minimum	Maximum		
*Oriochromianiloticus*	As	0.15	1.05	1.3E-05	8.0E-05	8.9E-05	5.6E-04	0.38	0.05
	Cd	0.07	0.49	4.6E-06	2.9E-05	3.2E-05	2.3E-04	0.29	0.04
	Co	0.12	0.84	4.4E-05	2.7E-04	3.0E-04	1.9E-03	1.61	0.23
	Cr	2	14	3.7E-05	2.3E-04	2.6E-04	1.6E-03	8.2E-02	0.01
	Cu	35	245	4.8E-05	3.0E-04	3.4E-04	2.1E-03	6.1E-03	8.7E-04
	Fe	56	392	1.8E-04	1.1E-03	1.2E-03	7.9E-03	1.4E-02	2.0E-03
	Mn	50	350	8.4E-05	5.3E-04	5.9E-04	3.7E-03	7.4E-03	1.1E-03
	Ni	0.35	2.45	3.7E-05	2.3E-04	2.6E-04	1.6E-03	0.47	0.07
	Pb	0.25	1.75	3.8E-05	2.4E-04	2.6E-04	1.7E-03	0.68	0.1
	Zn	70	490	1.1E-05	7.3E-05	8.0E-05	5.1E-04	7.3E-03	1.0E-04
*Coptodonkottae*	As	0.15	1.05	5.0E-06	3.2E-05	3.5E-05	2.2E-04	0.15	0.02
	Cd	0.07	0.49	5.1E-06	3.2E-05	3.6E-05	2.3E-04	0.32	0.05
	Co	0.12	0.84	4.2E-05	2.7E-04	2.9E-04	1.9E-03	1.55	0.22
	Cr	2	14	4.2E-05	2.6E-04	2.9E-04	1.8E-03	0.09	0.01
	Cu	35	245	4.0E-05	2.5E-04	2.8E-04	1.8E-03	5.1E-03	7.2E-04
	Fe	56	392	1.0E-04	6.6E-04	7.2E-04	4.6E-03	8.2E-03	1.2E-03
	Mn	50	350	8.3E-05	5.3E-04	5.8E-04	3.6E-03	7.4E-03	1.1E-03
	Ni	0.35	2.45	3.7E-05	2.3E-04	2.6E-04	1.6E-03	0.47	0.07
	Pb	0.25	1.75	3.8E-05	2.4E-04	2.6E-04	1.6E-03	0.67	0.1
	Zn	70	490	1.0E-05	6.4E-05	7.1E-05	4.5E-4	6.4E-4	9.1E-05

*PTWI* provisional tolerable weekly intake (mg/week/70 kg body wt.), *PTDI* permissible tolerable daily intake (mg/week/70 kg body wt.), *EWI* estimated weekly intake (mg/week/70 kg body wt.), *EDI* estimated dai1y intake (mg/week/70 kg body)

At a minimum ingestion rate of 15 g/day for both fish species, there is no heavy metal-associated exposure risk for both adults and children as the HQ values were all less than one (Table 3). It is unlikely that children would ingest fish at the rate of 95 g/day, but even if that happens, they are not likely to suffer non-cancer heavy metal-related complications due to consumption of both fish species in their lifetime because even at this high rate of consumption, the HQ values for all metals for children were below one. At an average fish consumption rate of 52 g/day, a Pb-associated non-cancer risk exists for adult individuals consuming both *O. niloticus* and *C. kottae* as indicated by the high HQ values (Table 3). This observation is also confirmed by the characterization factors for human toxicity of the various metals derived from the combination of human exposure through inhalation and ingestion and the USEtox effect factors related to human exposure to these metals. These factors indicated that As, Pb, and Cd had the highest toxicity potential as far as non-cancer-related effects are concerned, with 77.6, 17.3, and 12.4 cumulative cases, respectively, in a population exposed to a kg of metal emitted in a freshwater ecosystem. The results from this study show that continuous consumption of these two fish species harvested from the Barombi Kotto lake by adults may cause them to suffer from Pb-associated health risks during their lifetime. Lead has been associated with several health complications (ATSDR, 2021). According to Lam et al. (2007) and Shahid et al. (2012), Pb is implicated in blood-related disorders such as colic, constipation and anemia, high blood pressure, and decrease of hemoglobin production. Kidney, joints, reproductive and cardiovascular systems disorders have also been highlighted as some of the non-carcinogenic risks associated with exposure to Pb. Long-lasting injury to the central and peripheral nervous systems, loss of IQ, endocrine disruptive complications such as low sperm count, and loss of hearing are also highlighted as health complications caused by Pb exposure.

**Fig. 8:**
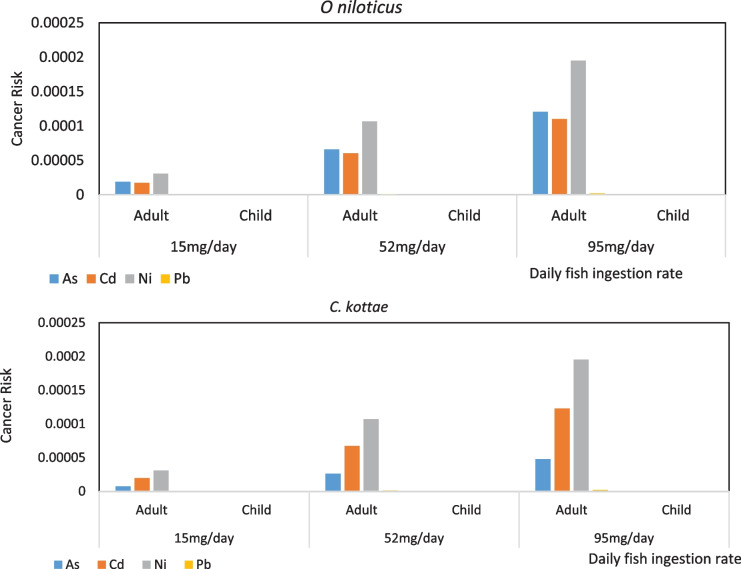
Cancer risk associated with ingestion of *O. niloticus* and *C. kottae* harvest from Lake Barombi Kotto

Using factors derived for the different heavy metals with the characterization factors of the USEtox Model and the two exposure pathways, the highest cancer-related toxicity potentials were recorded for As, Cd, Ni, and Pb with 8.6, 3.4 0.9, and 0.04 cases, respectively, per kg of emitted metal in freshwater ecosystems. The risk potential of the different metals was also observed in the non-cancer risk assessment using Risk Assessment guidance for superfund Volume I; Human Health Evaluation Manual Part A (Qu et al., 2012; USEPA, 2010). According to results obtained from these analyses, the likelihood that individuals consuming these two fish species harvested from Lake Barombi Kotto may develop cancer related to As, Cd, Ni, and Pb exposure was higher for adults than for children and for those consuming *O. niloticus* compared to those consuming *C. kottae* (Fig. 8).

Carcinogenic risks associated with exposure to these four metals for children ingesting these fish species were between 2.8 × 10^−11^ for Pb to 1.1 × 10^−7^ for Ni. These values were all below the unacceptable risk threshold for cancer risks (1 × 10^−4^). For adults, the cancer-related risk was slightly higher ranging from 3.2 × 10^−7^ for Pb to 2.0 × 10^−4^ for Ni with values for adults ingesting 52 g of either fish species per day having cancer risk values of 1.1 × 10^−4^ for Ni. The As-, Cd-, and Ni-associated cancer risks were significantly higher for adults ingesting O*. niloticus* at the rate of 95 g/day with risk values of 1.2 × 10^−4^, 1.1 × 10^−4^, and 1.9 × 10^−4^, respectively. Ingestion of *C. kottae* at the rate of 95 g/day presented Cd and Ni cancer-related risk to adults with cancer risk values of 1.2 × 10^−4^ and 2.0 × 10^−4^ for Cd and Ni, respectively.

Results from the health risk assessment have indicated that though the water quality of the lake is good, there exists the risk of heavy metal exposure through the ingestion of fish harvested from lakes in areas with no documented industrial activity. According to an FAO (2017) report, the main protein source for most Cameroonians is fish. The national average annual fish consumption in the country as of 2013 was 15.4 kg/person highlighting the significance of fish in the diet of the populace. Ingestion of fish contaminated with heavy metals, therefore, presents a health threat to this population.

## Conclusion

*O. niloticus* seems to accumulate more heavy metal than *C. kottae*. Results from this study have revealed that the bones of fish could present a hidden route of heavy metal exposure to individuals who ingest fish bones as they have been shown to accumulate significant amounts of heavy metals relative to the muscle. There is a need for monitoring and surveillance of activities in the area to ensure the existence of *C. kottae* found only in Lake Barombi Kotto and neighboring Lake Mboandong and to avoid the worsening of its IUCN conservational status in the ecosystem. While the consumption of fish harvested from relatively uncontaminated waters makes a very low contribution to the PTDI and PWTI, there exists the potential of heavy metal exposure for some heavy metals. In an area where there are few heavy industries that could release heavy metal-laden effluents into the lake, there still exists the risk of heavy metal pollution from subsistence agriculture and raw sewage and possibly natural processes like weathering. This could also highlight the fact that the role of sewage and wastewater effluents from unindustrialized regions as well as subsistence agriculture on heavy metal contamination of aquatic ecosystems are currently being undermined. The fast growth and reproductive rates of *O. niloticus* coupled with the other abiotic stressors already plaguing Lake Barombi Kotto may exacerbate and drive the few existing fish species to local extinction. Further studies are needed to investigate the effects of these metals on the reproductive cycles of these fish species and the toxicological implications of these metals on the community.

## Data Availability

All data supporting the results presented in the paper have been included in the Results section of the manuscript.

## References

[CR1] Aboud SJ, Nandini N (2009). Heavy metals analysis and sediment quality values in urban lakes. American Journal of Environmental Sciences.

[CR2] Adebayo I (2017). Determination of heavy metals in water, fish and sediment from Ureje water reservoir. Journal of Environmental & Analytical Toxicology.

[CR3] Ahmad AK, Al-Mahaqeri A (2015). Human health risk assessment of heavy metals in fish species collected from catchments of former tin mining. International Journal of Research Studies in Science, Engineering and Technology.

[CR4] Algül F, Beyhan M (2020). Concentrations and sources of heavy metals in shallow sediments in Lake Bafa. Turkey. Scientific Reports.

[CR5] ATSDR. (2021). *Toxic substances portal. toxicological profiles: ATSDR; 2016 [assessed in November 2021).* http://www.atsdr.cdc.gov/toxprofiles/index.asp

[CR6] Authman M, Zaki M, Khallaf E, Abbas H (2015). Use of fish as bio-indicator of the effects of heavy metals pollution. Journal of Aquaculture Research and Development.

[CR7] Awachat AR, Salkar VD (2017). Ground water quality assessment through WQIs. International Journal of Engineering Research and Technology..

[CR8] Awo M, Tabot P, Fonge B (2021). Tree species composition and diversity in the riparian forest of lake Barombi Kotto. Cameroon. American Journal of Plant Sciences.

[CR9] Awo ME (2020). Water quality of the volcanic crater lake, Lake Barombi Kotto. Cameroon. African Journal of Aquatic Science..

[CR10] Awo ME, Fonge BA, Tabot PT (2019). Ecosystem services and perception of water quality of Lake Barombi Kotto, Cameroon. International Journal of Trends in Scientific Research & Development.

[CR11] Ayanda IO, Dedeke GA, Ekhator UI, Etiebet MK (2018). Proximate composition and heavy metal analysis of three aquatic foods in Makoko River, Lagos. Nigeria. Journal of Food Quality.

[CR12] Bassem, S. (2020). Water pollution and aquatic biodiversity. *Biodiversity International Journal 4*(1), 10 - 16. 10.15406/bij.2020.04.00159

[CR13] Bawuro AA, Voegborlo RB, Adimado AA (2018). Bioaccumulation of heavy metals in some tissues of fish in Lake Geriyo, Adamawa State, Nigeria. Journal of Environmental and Public Health.

[CR14] Boehnert S, Ruiz Soto S, Fox BRS, Yokoyama Y, Hebbeln D (2020). Historic development of heavy metal contamination into the Firth of Thames New Zealand. Geo-Marine Letters.

[CR15] Bowes, M. J., Jarvie, H. P., Halliday, S. J., Skeffington, R. A., Wade, A. J., Loewenthal, M., Gozzard, E., Newman, J. R., & Palmer-Felgate, E. J. (2015). Characterising phosphorus and nitrate inputs to a rural river using high-frequency concentration–flow relationships. *Science of The Total Environment*, *511*, 608–620. 10.1016/j.scitotenv.2014.12.086 10.1016/j.scitotenv.2014.12.086 25596349

[CR16] Bunce, J. T., Ndam, E., Ofiteru, I. D., Moore, A., & Graham, D. W. (2018). A review of phosphorus removal technologies and their applicability to small-scale domestic wastewater treatment systems [Review]. *Frontiers in Environmental Science*, *6(8)*. https://www.frontiersin.org/article/10.3389/fenvs.2018.00008

[CR17] Calvo MS, Uribarri J (2013). Public health impact of dietary phosphorus excess on bone and cardiovascular health in the general population. American Journal of Clinical Nutrition.

[CR18] Campbell SJ, Stothard JR, O'Halloran F, Sankey D, Durant T, Ombede DE, Chuinteu GD, Webster BL, Cunningham L, LaCourse EJ, Tchuem-Tchuenté LA (2017). Urogenital schistosomiasis and soil-transmitted helminthiasis (STH) in Cameroon: An epidemiological update at Barombi Mbo and Barombi Kotto crater lakes assessing prospects for intensified control interventions. Infectious Disease and Poverty.

[CR19] Carpenter SR, Stanley EH, Vander Zanden MJ (2011). State of the world’s freshwater ecosystems: Physical, chemical, and biological changes. Annual Review of Environment and Resources.

[CR20] Chapman P, Wang F, Janssen C, Persoone G, Allen H (2011). Ecotoxicology of metals in aquatic sediments: Binding and release, bioavailability, risk assessment, and remediation. Canadian Journal of Fisheries and Aquatic Sciences.

[CR21] Cicero-Fernández D, Peña-Fernández M, Expósito-Camargo JA, Antizar-Ladislao B (2017). Long-term (two annual cycles) phytoremediation of heavy metal-contaminated estuarine sediments by Phragmites australis. New Biotechnology.

[CR22] Dallinger R, Prosi F, Segner H, Back H (1987). Contaminated food and uptake of heavy metals by fish: A review and a proposal for further research. Oecologia.

[CR23] Dhingra R, Sullivan LM, Fox CS, Wang TJ, D'Agostino RB, Gaziano JM, Vasan RS (2007). Relations of serum phosphorus and calcium levels to the incidence of cardiovascular disease in the community. Archives of Internal Medicine.

[CR24] El-Moselhy, K. M., Othman, A. I., Abd El-Azem, H., & El-Metwally, M. E. A. (2014). Bioaccumulation of heavy metals in some tissues of fish in the Red Sea, Egypt. *Egyptian Journal of Basic and Applied Sciences*, *1*(2), 97–105. 10.1016/j.ejbas.2014.06.001

[CR25] Ersoy, Z., Brucet, S., Bartrons, M., & Mehner, T. (2019). Short-term fish predation destroys resilience of zooplankton communities and prevents recovery of phytoplankton control by zooplankton grazing. *Plos One*, *14*(2), e0212351. 10.1371/journal.pone.0212351 10.1371/journal.pone.0212351 PMC6377254 30768619

[CR26] FAO. (2017). *Fisheries and Aquaculture Country Profiles; The Republic of Cameroon.* (Part 1 Statistics and Main indicators. https://www.fao.org/fishery/en/facp/cmr?lang=fr

[CR27] FAO, WHO.  (2010). Summary report of the seventy-third meeting of lECFA".

[CR28] Fawole, O., Akinloye, O., & Tolulope, A. (2007). Proximate and mineral composition in some selected fresh water fishes in Nigeria. *Journal of Food Safety*, *9*, 52 - 52.

[CR29] Fernandez-Maestre R, Johnson-Restrepo B, Olivero-Verbel J (2018). Heavy metals in sediments and fish in the Caribbean coast of Colombia: Assessing the environmental risk. International Journal of Environmental Research.

[CR30] Fitton, J. G., & Dunlop, H. M. (1985). The Cameroon line, West Africa, and its bearing on the origin of oceanic and continental alkali basalt. *Earth and Planetary Science Letters*, *72*(1), 23–38. 10.1016/0012-821X(85)90114-1

[CR31] Fonkou T, Agendia P, Kengne IM, Akoa A, Focho D, Nya J, Dongmo F (2005). Heavy metal concentrations in some biotic and abiotic components of the Olezoa wetland complex (Yaoundé–Cameroon, West Africa). Water Quality Research Journal of Canada.

[CR32] Froese, R., & Pauly, D. (2015). *Tilapia kottae Lönnberg, 1904. FishBase.* http://www.fishbase.org/summary/8919

[CR33] Gall J, Boyd R, Rajakaruna N (2015). Transfer of heavy metals through terrestrial food webs: A review. Environmental Monitoring and Assessment.

[CR34] Gbogbo, F., Arthur-Yartel, A., Bondzie, J. A., Dorleku, W. -P., Dadzie, S., Kwansa-Bentum, B., Ewool, J., Billah, M. K., & Lamptey, A. M. (2018). Risk of heavy metal ingestion from the consumption of two commercially valuable species of fish from the fresh and coastal waters of Ghana. *Plos One*, *13*(3), e0194682. 10.1371/journal.pone.0194682 10.1371/journal.pone.0194682 PMC5865748 29570730

[CR35] George M, Ngole-Jeme VM (2022). An evaluation of the Khubelu wetland and receiving stream water quality for community use. Water.

[CR36] Hauschild MZ, Huijbregts M, Jolliet O, Macleod M, Margni M, van de Meent D, Rosenbaum RK, McKone TE (2008). Building a model based on scientific consensus for life cycle impact assessment of chemicals: The search for harmony and parsimony. Environmental Science & Technology.

[CR37] Hossain MB, Tanjin F, Rahman MS, Yu J, Akhter S, Noman MA, Sun J (2022). Metals bioaccumulation in 15 commonly consumed fishes from the lower Meghna river and adjacent areas of Bangladesh and associated human health hazards. Toxics.

[CR38] Jezierska B, Witeska M (2006). 2006//). The metal uptake and accumulation in fish living in polluted waters. Soil and Water Pollution Monitoring, Protection and Remediation.

[CR39] Kangabam D, Bhoominathan R, Devi S, Suganthi K, Munisamy G (2017). Development of a water quality index (WQI) for the Loktak Lake in India. Applied Water Science.

[CR40] Kar S, Maity JP, Jean JS, Liu CC, Liu CW, Bundschuh J, Lu HY (2011). Health risks for human intake of aquacultural fish: Arsenic bioaccumulation and contamination. Journal of Environmental Science and Health Part A Toxic/ Hazardous Substances and Environmental Engineering.

[CR41] Krivokapić M (2021). Study on the evaluation of (heavy) metals in water and sediment of Skadar Lake (Montenegro), with BCF assessment and translocation ability (TA) by Trapa natans and a review of SDGs. Water.

[CR42] Kwok CK, Liang Y, Leung SY, Wang H, Dong YH, Young L, Giesy JP, Wong MH (2013). Biota-sediment accumulation factor (BSAF), bioaccumulation factor (BAF), and contaminant levels in prey fish to indicate the extent of PAHs and OCPs contamination in eggs of waterbirds. Environmental Science and Pollution Research International.

[CR43] Lam TV, Agovino P, Niu X, Roché L (2007). Linkage study of cancer risk among lead-exposed workers in New Jersey. Science of the Total Environment.

[CR44] Lin, T. -C., Yang, C. -R., & Chang, F. -H. (2007). Burning characteristics and emission products related to metallic content in incense. *Journal of Hazardous Materials*, *140*(1), 165–172. 10.1016/j.jhazmat.2006.06.052 10.1016/j.jhazmat.2006.06.052 16973266

[CR45] Malik, D., Sharma, A., Sharma, A., Thakur, R., & Sharma, M. (2020). A review on impact of water pollution on freshwater fish species and their aquatic environment. In Advances in environmental pollution management: wastewater impacts and treatment technologies (pp. 10–28). 10.26832/aesa-2020-aepm-02

[CR46] Manga, V. E., Suh, C. E., Agyingi, C. M., & Shemang, E. M. (2013). Mineralogy and geochemistry of soils developed along the slopes of Mt. Cameroon, West Africa. *Journal of African Earth Sciences*, *81*, 82–93. 10.1016/j.jafrearsci.2013.01.008

[CR47] Moe S, Drüeke T, Cunningham J, Goodman W, Martin K, Olgaard K, Ott S, Sprague S, Lameire N, Eknoyan G (2006). Definition, evaluation, and classification of renal osteodystrophy: A position statement from Kidney Disease: Improving Global Outcomes (KDIGO). Kidney International.

[CR48] Ngole-Jeme, V. M., & Fantke, P. (2017). Ecological and human health risks associated with abandoned gold mine tailings contaminated soil [Article]. *Plos One*, *12*(2), Article ARTN e0172517. 10.1371/journal.pone.0172517 10.1371/journal.pone.0172517 PMC5319768 28222184

[CR49] Osman, H., Suriah, A. R., & Law, E. C. (2001). Fatty acid composition and cholesterol content of selected marine fish in Malaysian waters. *Food Chemistry*, *73*(1), 55–60. https://eurekamag.com/research/003/445/003445157.php

[CR50] Pinzón-Bedoya CH, Pinzón-Bedoya ML, Pinedo-Hernández J, Urango-Cardenas I, Marrugo-Negrete J (2020). Assessment of potential health risks associated with the intake of heavy metals in fish harvested from the largest estuary in Colombia. International Journal of Environmental Research and Public Health.

[CR51] Pouokam GB, Foudjo BUS, Samuel C, Yamgai PF, Silapeux AK, Sando JT, Atonde GF, Frazzoli C (2017). Contaminants in foods of animal origin in Cameroon: A one health vision for risk management “from Farm to Fork”. Frontiers in Public Health.

[CR52] Pouomogne, V., & Pemsl, D. (2008). Recommendation domains for pond aquaculture country case study: Development and status of freshwater aquaculture in Cameroon. The WorldFish Center. Penang Malaysia 60 pages.

[CR53] Prasad B, Maiti D (2016). Comparative study of metal uptake by Eichhornia crassipes growing in ponds from mining and nonmining areas—A field study. Bioremediation Journal.

[CR54] Qadir A, Malik RN (2011). Heavy metals in eight edible fish species from two polluted tributaries (Aik and Palkhu) of the River Chenab. Pakistan. Biological Trace Element Research.

[CR55] Qu, C. -S., Ma, Z. -W., Yang, J., Liu, Y., Bi, J., & Huang, L. (2012). Human exposure pathways of heavy metals in a lead-zinc mining area, Jiangsu Province, China. *Plos One*, *7*(11), e46793. 10.1371/journal.pone.0046793 10.1371/journal.pone.0046793 PMC3496726 23152752

[CR56] Rosenbaum RK, Bachmann TM, Gold LS, Huijbregts MAJ, Jolliet O, Juraske R, Koehler A, Larsen HF, MacLeod M, Margni M, McKone TE, Payet J, Schuhmacher M, van de Meent D, Hauschild MZ (2008). USEtox—the UNEP-SETAC toxicity model: Recommended characterisation factors for human toxicity and freshwater ecotoxicity in life cycle impact assessment. The International Journal of Life Cycle Assessment.

[CR57] Shahid M, Pinelli E, Dumat C (2012). Review of Pb availability and toxicity to plants in relation with metal speciation; role of synthetic and natural organic ligands. Journal of Hazardous Materials.

[CR58] Tonelli M, Sacks F, Pfeffer M, Gao Z, Curhan G (2005). Relation between serum phosphate level and cardiovascular event rate in people with coronary disease. Circulation.

[CR59] Tüzen M (2003). Determinaton of heavy metals in fish samples of the Middle Black Sea (Turkey) by graphite furnace atomic absorption spectrometry. Food Chemistry.

[CR60] USEPA. (2010). *Risk assessment guidance for superfund Volume I. Human health evaluation manual (Part A).* . Washington, DC.. Office of Emergency and Remedial Response.

[CR61] USEPA. (2017). *USEPA, IRIS. United States, Environmental Protection Agency, Integrated Risk Information System.* http://www.epa.gov/iris/subst

[CR62] Weber, P., Behr, E. R., Knorr, C. D. L., Vendruscolo, D. S., Flores, E. M. M., Dressler, V. L., & Baldisserotto, B. (2013). Metals in the water, sediment, and tissues of two fish species from different trophic levels in a subtropical Brazilian river. *Microchemical Journal*, *106*, 61–66. 10.1016/j.microc.2012.05.004

[CR63] Welcomme RL (2011). An overview of global catch statistics for inland fish. ICES Journal of Marine Science.

[CR64] Xu M, Wang Z, Duan X, Pan B (2014). Effects of pollution on macroinvertebrates and water quality bio-assessment. Hydrobiologia.

